# Biocompatibility of novel albumin-aldehyde surgical adhesive

**DOI:** 10.1038/s41598-022-16853-5

**Published:** 2022-07-26

**Authors:** Lukasz Szymanski, Kamila Gołaszewska, Anna Wiatrowska, Monika Dropik, Patrycja Krakowiak, Justyna Małkowska, Damian Matak

**Affiliations:** 1 Department of Molecular Biology, Institute of Genetics and Animal Biotechnology, Polish Academy of Science, Postępu 36A, 05-552 Magdalenka, Poland; 2 European Biomedical Institute, Nalkowskiej 5, 05-410 Jozefow, Poland

**Keywords:** Preclinical research, Biomaterials, Materials for devices, Biomaterials

## Abstract

Many medical procedures could benefit from the use of tissue sealants which allow for reduced surgery time, limited blood loss, easier tissue handling, and fewer postoperative complications. The safety and biocompatibility of surgical sealants are of paramount importance therefore, the aim of this study is to investigate the biocompatibility of NE’X Glue Surgical Adhesive. Chemical characterization (VOC and elements), cytotoxicity (MEM elution), genotoxicity (AMES and MLA), endotoxin contamination, sensitization potential, intracutaneous reactivity, acute and subchronic systemic toxicity with implantation as well as pyrogenicity were evaluated to investigate the biocompatibility of the NE’X Glue Surgical Adhesive. Studies were conducted according to ISO 10993 standards. The biocompatibility requirements with accordance to ISO 10993-1 for NE'X Glue were met. In vitro studies showed that NE'X Glue surgical adhesive is non-cytotoxic and non-mutagenic. Also, in vivo studies demonstrated that NE'X Glue shows no signs of toxicity, has no pyrogenic potential, and is non-sensitizing and non-irritating. The chemical characterization showed that no compounds were identified above Analytical Evaluation Threshold (AET), and no elements with concentrations higher than element-specific PDE (µg/day) were detected. NE'X Glue Surgical Adhesive is a versatile and promising new surgical sealant with a wide range of potential applications and very good biocompatibility.

## Introduction

Each year, countless medical procedures are performed to allow for wound closure, stop bleeding and prevent leaks. Conventional methods of achieving hemostasis include the use of staples, sutures, clips, and electrocoagulation. Even though these methods work well for most procedures, in more challenging applications, they do not perform that well^1^. Sutures are most commonly used, but they are also characterized by many disadvantages. Their placement may be challenging and time-consuming, induces damage, and the immunological reaction of the tissue increases the chance of microbial infection. Therefore, many medical procedures could benefit from the use of tissue sealants which allow for reduced surgery time, limited blood loss, easier tissue handling, and fewer postoperative complications. Moreover, the use of adhesives lowers or eliminates the localized load stress between fractured surfaces^2,3^. Surgical adhesives are emerging to be a gold standard in clinical practice as an adjunct to standard methods of achieving hemostasis to prevent air and liquid leakages during surgeries. Sealant's physical properties and adhesion strength to seal the wound area without limiting the tissue function and movement are key factors in their successful implementation in clinical practice. Optimally, surgical sealants should be biodegradable without causing an inflammatory response, polymerize well in a moist environment, possess satisfactory adhesive strength, and meet biocompatibility requirements with no or minimal tissue toxicity^4–6^.

As defined in ISO 10993-1, biocompatibility is an ability of a medical device or material to perform with an appropriate host response in a specific application^7^. The ultimate goal of biocompatibility testing is to reduce the risks associated with medical devices within limits set by the relevant legislation, but a product's biocompatibility test results may be at either end of the permitted spectrum. The degree of biocompatibility correlates with the risk of clinical use of the medical device, so in other words, the risk of occurrence of adverse reactions is inversely proportional to the biocompatibility testing results. The use of non-biocompatible medical devices can lead to severe health consequences, including systemic toxicity and death. It is particularly important in the case of class III medical devices, which are associated with high risk. The safety and biocompatibility of surgical sealants are of paramount importance therefore in the present study, the biocompatibility of NE'X Glue Surgical Adhesive according to the ISO 10993 has been investigated.

## Study product

**Figure 1:**
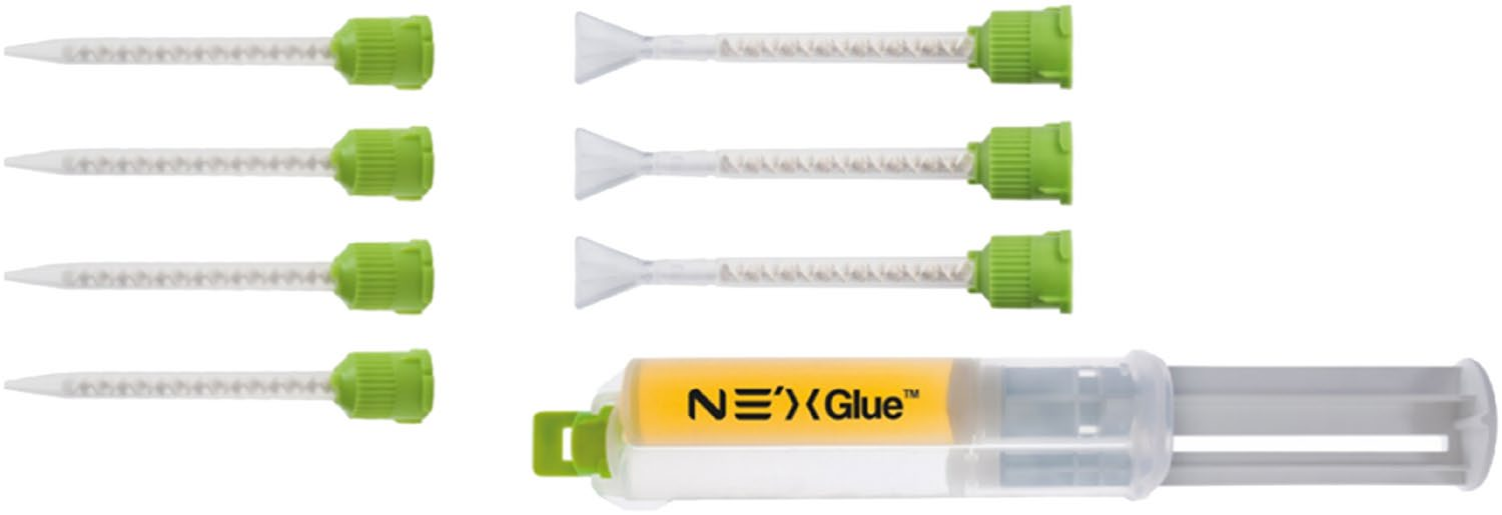
Photo of NE’X Glue.

NE'X Glue is a two-component surgical adhesive composed of purified albumin solution and aldehyde solution, which is sterilized via gamma-irradiation (Fig. 1). The solutions are dispensed by a controlled delivery system and applicator tips which are sterilized via EO. Double-chambered syringe and applicator tips are designed to provide precise and reproducible mixing of components during application. This surgical adhesive begins to polymerize around 20 s and becomes fully polymerized within 2 min after application. During the application of NE'X Glue, aldehyde solution and albumin solution start to mix within the applicator tip. Upon contact with tissue at the application site, aldehyde solution crosslinks with albumin solution and tissue proteins creating a seal. The bondage between aldehyde, albumin, and tissue proteins is known as a covalent bond. During the polymerization process, lysine side chains of proteins are crosslinked (covalently bonded) with the aldehyde.

## Material and methods

The methods used in this study were previously extensively described in our previous publication^8^. Therefore, only a brief description is provided in the Material and Methods sections wherever possible. Detailed information is present in “Supplementary materials”. Cell lines were purchased from ATCC, reagents for cell culture were purchased from Thermo Fisher Scientific, Poland, and all chemical compounds were purchased from Sigma, Poland, unless otherwise indicated.

### Ethics committee

All studies were conducted in accordance to the guidelines of the Declaration of Helsinki, approved by the I Local Ethics Committee in Warsaw, and conducted under the protocol codes 738/2018, 879/2019, and 864/2019. The experimental protocols were approved by both I Local Ethical Committee for Animal Experiments in Warsaw and European Biomedical Institute.

### Statistical analysis

All results were presented as the mean ± standard deviation (SD). Subacute toxicity combined with implantation results were analyzed using two-tailed heteroscedastic T-test. GraphPad Prism software (version 9.3.1; GraphPad Software, Inc., La Jolla, CA, USA) was used for all evaluations. p < 0.05 was considered statistically significant.

### Extraction

Extraction conditions were chosen based on the ISO 10993-12 and study appropriate ISO norms^9^. Briefly, the extractions were prepared by incubating the test material with a suitable extraction medium at 37 ± 1 °C for 72 ± 2 h unless otherwise indicated. 37 degrees were chosen because higher temperatures may cause degradation of the tested sample due to high protein content. The extraction volume was derived from Table 1—Standard surface areas and extract liquid volumes, ISO 10993-12, and determined at 0.2 g/mL^9^. The extracts were not centrifuged, filtered, or otherwise altered prior to dosing. The extract was clear without the presence of any particulates. The extracts were used within 24 h of preparation.

**Table 1:** Method of application of test samples.

Group	Induction		Challenge topical application
	Intradermal injection [pair of 0.1 ml injection]	Topical application	
Sodium chloride extract 10 animals	(a) 50:50 (v:v) stable emulsion of Freud's complete adjuvant mixed with appropriate test sample extract or solvent control (b) Undiluted appropriate test sample extract or solvent control (c) Undiluted test samples extract emulsified in a 50:50 (v:v) stable emulsion of Freund's complete adjuvant and appropriate solvent control (50%)	Sodium chloride extract	Right costa region: appropriate test sample extract 50:50 (v:v) Left costa region: appropriate solvent control
Cottonseed oil extract 10 animals		Cottonseed oil extract	
Solvent control: sodium chloride 5 animals		Sodium chloride	
Solvent control: cottonseed oil 5 animals		Cottonseed oil	

### Chemical characterization

According to the ISO 10993-18, a semi-quantitative VOC (volatile organic compound) analysis was performed in NE'X Glue water extract^10^. In addition, a quantitative analysis of elements concentration in NE'X Glue water extract was performed.

Assuming an uncertainty factor of 2, NE'X Glue per patient usage, and the Threshold of Toxicological Concert of 1.5 µg/day, the AET was calculated to be 0.015 µg/mL.

Additional data is available in the “Supplementary materials”.

### Cytotoxicity

Cytotoxicity was evaluated quantitatively using the MEM Elution method based on the ISO 10993-5 and ISO 10993-12 and was described before^8,9,11^. Briefly, NE'X Glue was extracted in single strength MEM for 24 ± 1 h at 37 ± 1 °C using a 0.2 g/ml extraction ratio. Following the extraction, 600 µL of extracts were dosed to triplicate monolayers of L929 cells and incubated in the presence of 5 ± 0.1% CO_2_, 95% humidity for 24 ± 1 h. DMSO was used as the positive control and HDPE extract was used as negative control. Afterward, 100 µl of freshly prepared staining solution (mixture of Trypan Blue solution with single strength MEM in 1:1 ratio) was dispended in each well. Finally, cytotoxicity was assessed by microscopic observations according to Table 1 included in ISO 10993-5.

### Genotoxicity

Extraction of NE'X Glue for genotoxicity studies.

The amount of extractables was assessed by a pre-experiment “Determination of Extractables” according to ISO 10993-3^12^. Based on the results, Method C—extraction according to ISO 10993-12 was chosen^9^. The extraction was conducted using an appropriate extraction vehicle.

### Mouse Lymphoma Assay (MLA)

As described previously, based on the ISO 10993-3, ISO 10993-12, ISO 10993-33, and OECD Test No 490, the NE'X Glue genotoxicity was evaluated using Mouse Lymphoma Assay^9,12–15^. Additional data is available in the “Supplementary materials”.

### Bacterial Reverse Mutation Test-AMES

Genotoxicity of NE'X Glue was evaluated using commercially available Bacterial Reverse Mutation Test AMES Penta 2 (Xenometrix) according to ISO 10993-3, ISO 10993-12, ISO 10993-33, and OECD Test No. 471^9,12,14,16^. Additional data is available in the “Supplementary materials”.

### Endotoxins

Endotoxins were measured using Pierce Chromogenic Endotoxin Quant K, which is in regard to 85. Bacterial Endotoxin Test, U.S. Pharmacopoeia^17^. The NE'X Glue was extracted in water for injection using an extraction ratio of 0.2 g/ml. The standard curve was prepared according to the manufacturer's instruction (R^2^ = 0.9946). Internal validation of the experiment was performed by spiking the samples with 0.5 EU/ml of endotoxin. The unspiked and spiked samples were assayed to determine the respective endotoxin concentrations. For the test to be valid, the difference between the two calculated endotoxin values should equal the known (0.5 EU/ml) concentration of the spike ± 25%.

### Sensitization

The sensitization potential of the NE'X Glue was analyzed according to the ISO 10993-10 using the Guinea Pig Maximization Test (GPMT)^18^. Briefly, NE'X Glue was extracted using sodium chloride and cottonseed oil. Then, 30 male guinea pigs (Dunkin-Hartley) were randomly assigned to study groups (10 animals each) and solvent control groups (5 animals each). Before testing began, the fur was removed by shaving approximately 50 cm^2^ on the back of the animals.

#### Intradermal induction phase

Three pairs of 0.1 ml intradermal injections were made in the interscapular region of each animal, on each side of the midline (injection sites A, B, C). Sites of the injections were marked with a permanent skin marker.

#### Topical induction phase

6 days after the start of the treatment Sodium Dodecyl Sulfate in Vaseline was massaged into the skin at the injection site B. 24 h later, 7 days from the initial intradermal induction, 0.5 ml of the test sample extract or solvent control was applied to each animal. Application sites were covered in dressings for 48 h.

#### Challenge phase

At 12 days after completion of the topical induction phase—0.5 ml of test samples extracts were applied to the right costa region. Appropriate solvent controls were applied to the left costa region of each animal. Application sites were covered in dressings for 24 h.

The summarized methodology of application is presented in Table 1.

24 ± 2 and 48 ± 2 h after removing the patches, all treated and control animals were visually evaluated for a skin reaction. The intensity of erythema and/or oedema were evaluated according to the Magnusson and Kligman scale.

Magnusson and Kligman grades of 1 or greater in the test group indicate sensitization, assuming grades of less than 1 are seen in control animals. If grades of 1 or greater are noted in control animals, then the reactions of test animals that exceed the most severe reaction in control animals are presumed to be due to sensitization.

Additional data is available in the “Supplementary materials”.

### Intracutaneous reactivity

The study was conducted according to ISO 10993-10^18^. The test article was extracted using Sodium Chloride and Cottonseed Oil as described above. The test was performed on New Zealand rabbits. Additional data is available in the “Supplementary materials”.

### Acute systemic toxicity

The study was conducted according to ISO 10993-10^18^. Four groups of 5 BALB/c mice were injected with 50 ml/kg of Sodium Chloride extract, Cottonseed Oil extract, and the polar and non-polar solvent controls. Polar and non-polar extracts and solvents controls were injected intraperitoneal. Animals underwent a clinical examination and were weighted 24 ± 2 h, 48 ± 2 h, 72 ± 2 h after injection. 72 ± 2 h after injection, animals were euthanized.

### Subchronic toxicity combined with implantation

Based on ISO 10993-6 and ISO 10993-11, NE'X Glue was evaluated for subchronic toxicity combined with implantation using BioGlue as reference material^19,20^.

Before the treatment, the fur on the each rat’s (Wistar) back was clipped over the test area, avoiding mechanical irritation and trauma. The place of implantation was disinfected with iodine solution. Procedure was performed under general anesthesia using isoflurane. If necessary, animals were subcutaneously injected with butorphanol (2 mg/kg). During the surgery, incision was made on the skin in a paraspinal line to create separate pockets in subcutaneous tissue. Implants were placed on both flanks of the animal at equal intervals. 8 implants of either tested or control article per rat were implanted. Based on the maximal volume of NE'X Glue designed to be used per patient and human statistical weight (60 kg), each animal was implanted with 8 0.04 ml implants. Based on the weight of the animals, the evaluated quantity of the tested product is more than 10× the dose used in a clinical setting. Wounds were closed using non-resorbable threads. Each animal was injected subcutaneously with meloxicam (1 mg/kg) for three days after implantation. Animals were housed separately for a week until the wound healed, and then they were put together. Animals were weighed and observed 1, 2, 3, 7, 14, 21, 28, 35, 42, 49, 56, 63, 70, 77, 84, 90 days after implantation.

After the observation period, urine and blood samples were collected. Routine test such as hematology and clinical chemistry were conducted on all animals. Briefly, animals were anesthetized with Ketamine/Xylazine (100 mg/kg—Ketamine, 10 mg/kg—Xylazine), and blood was drawn into K2-EDTA tubes hematology and heparin for clinical chemistry. Total WBC, Hb, RBC, PCV, reticulocytes, and thrombocytes were determined with a hematology analyzer. ALP, ALAT, ASAT, GGT, glucose plasma concentration, total protein, albumin, urea, creatinine, total cholesterol, total bilirubin, phospholipids, triglycerides, Cl^−^, Ca^2+^, Na^+^, K^+^, and inorganic phosphate were determined using a biochemical analyzer.

Additional data is available in the “Supplementary materials”.

### Pyrogenicity

#### Rabbit selection

Negative pyrogen test was performed on all rabbits (New Zealand) used in the study with 14 days preceding the assay (each rabbit had a rest period of a minimum of 3 days after negative pyrogen pretest).

#### Determination of the initial temperature

Temperature of each rabbit was recorded every 30 min for 90 min before injection using thermometric rectal probe inserted at not less than 7.5 cm but not more than 9 cm. The rabbits that showed a temperature variation of two successive readings higher than 0.2 °C during the initial temperature determination or a temperature higher than 39.6 °C or lower than 38.2 °C were excluded from the study. The initial temperature of each rabbit was determined as the mean of two temperatures recorded at intervals of 30 min before the injection. In the group, the difference between the three initial temperatures did not exceed 1 °C.

#### Rabbit injection and follow up

After extraction, the tested solution was equilibrated at 38.5 °C and injected intravenously through the marginal ear vein at a dose of 10 ml/kg of body weight. The temperature of each rabbit was recorded every 30 min for 3 h after injection. The maximum rise of each rabbit was determined at the end of the test. Criteria of acceptance for the pyrogenicity test are presented in the “Supplementary materials”.

### Ethics approval and consent to participate

The study was conducted according to the guidelines of the Declaration of Helsinki and approved by the I Local Ethics Committee in Warsaw protocol codes 738/2018, 879/2019, and 864/2019. All animal research methods were planned and reported in accordance with ARRIVE guidelines.

## Results

### Chemical characterization

Extraction condition determination for exhaustive extraction of N'EX Glue showed that hexane and isopropanol cause product degradation and change of vehicle color. Therefore, according to the ISO 10993-18, only water extract was analyzed.

In the study, no VOCs above AET were identified.

No elements above the limit of detection were identified. A comparison of LODs and Parenteral PDE is presented in Table 2.

**Table 2:** ICP MS results of NE'X glue.

Analyzed element	Limit of detection (LOD) (µg/l)	Parenteral PDE (µg/day)
Cd	1	2
Pb	2.5	5
As	30	15
Hg	1	3
Co	2.5	5
V	24	10
Ni	10	20
Tl	5	8
Au	5	100
Pd	5	10
Ir	5	10
Os	5	10
Rh	5	10
Ru	5	10
Se	10	80
Ag	5	10
Pt	5	10
Li	10	250
Sb	10	90
Ba	10	700
Mo	10	1500
Cu	130	300
Sn	10	600
Cr	60	1100

### Cytotoxicity

NE'X Glue cell culture medium extract showed no cytotoxic potential to L-929 mouse fibroblasts in the MEM Elution assay. The suitability of the test system was confirmed based on the cellular response observed in the positive and negative controls.

**Figure 2:**
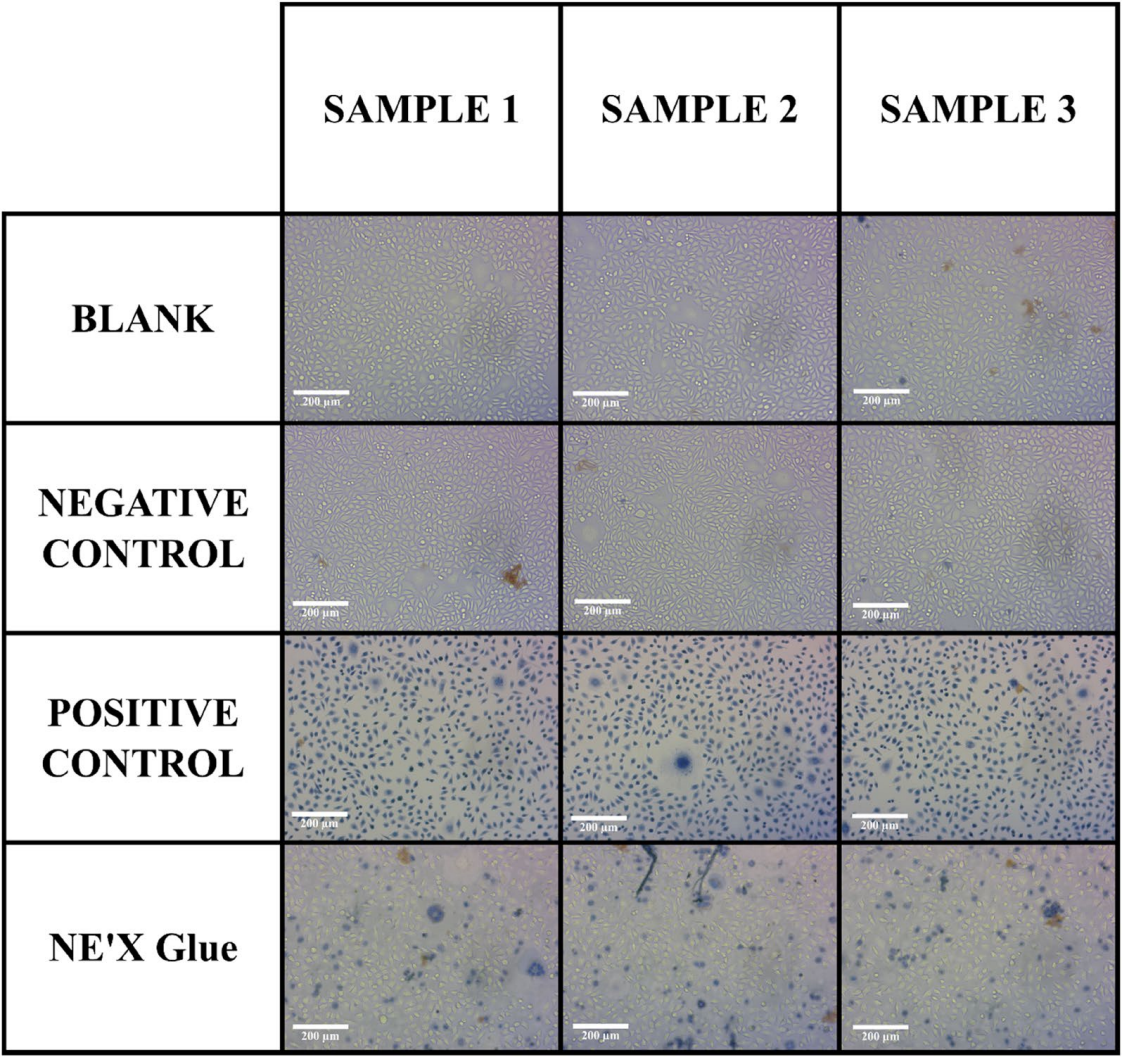
Images of cells after exposure to surgical adhesive extracts, negative and positive controls in the MEM elution study.

Cytotoxicity test results are presented in Table 3 and Fig. 2.

**Table 3:** Results of cytotoxic potential assessment.

Sample	Grade	System suitability
Blank	0	Valid
Negative control	0	Valid
Positive control	4	Valid
NE'X glue	2	No cytotoxic potential

### Genotoxicity (MLA and AMES)

#### AMES

Every bacterial strain that was used in the test, both with and without S9 fraction, passed internal quality controls. N'EX Glue showed an unclear mutagenic effect only when exposed to the TA1535 strain with the presence of the S9 fraction. Results are presented in Tables 4 and 5.

**Table 4:** AMES assay results.

Strain	Without S9			With S9		
	Baseline	Fold increase over baseline	Binomial B-value	Baseline	Fold increase over baseline	Binomial B-value
TA98	1.82	0.55	0.86	6.46	0.83	0.99
TA1535	2.49	0.54	0.63	1	1	0.98
TA1537	1	0.67	0.92	8.29	0.16	0.01
*E. coli* uvrA[pKM101]	3.49	0.19	0.03	13.44	1.09	1

**Table 5:** AMES overall results.

Strain	Mutagenic data points		Overall result for sample	Solvent control		Positive control	
	W/o S9	With S9		W/o S9	With S9	W/o S9	With S9
TA98	No	No	Probably not mutagenic	PASS	PASS	PASS	PASS
TA1535	No	No	Probably not mutagenic	PASS	PASS	PASS	PASS
TA1537	No	No	Probably not mutagenic	PASS	PASS	PASS	PASS
*E. coli* uvrA[pKM101]	No	No	Probably not mutagenic	PASS	PASS	PASS	PASS

Analyzed and summarized data is presented in Table 5 below. No precipitation or toxicity was observed in this study.

#### Mouse Lymphoma Assay (MLA)

If the MF is above the Global Evaluation Factor of 126 (× 10^–6^) over the negative control the sample is considered mutagenic. The acceptance criteria for MLA have been previously described^13^. No toxicity or precipitation was observed in this study. Results are presented in Tables 6, 7, 8, 9, 10 and 11.

**Table 6:** Toxicity data, 4 h exposure, without metabolic activation.

Sample	Number of seeded cells (× 10^5^)	Number of cells after 24 h treatment (× 10^5^)	Number of cells after 48 h treatment (× 10^5^)	Total suspension growth	Relative suspension growth (RSG) (%)	Plating efficiency (%)	Relative plating efficiency (RPE) (%)	Relative total growth (RTG) (%)
NC1	3.00	11.02	9.03	16.59	100.00	93.59	100.00	100.00
NC2	3.00	10.56	9.11	16.03				
PC	3.00	8.62	7.67	11.02	67.56	65.80	70.31	47.50
NE’X Glue1	3.00	10.34	8.84	15.23	93.41	98.04	104.76	97.85
NE’X Glue2	3.00	10.11	8.95	15.08	92.47	89.30	95.42	88.24

*PC* positive control, *NC* negative control.

**Table 7:** Toxicity data, metabolic activation, 4 h exposure.

Sample	Number of seeded cells (× 10^5^)	Number of cells after 24 h treatment (× 10^5^)	Number of cells after 48 h treatment (× 10^5^)	Total suspension growth	Relative suspension growth (RSG) (%)	Plating efficiency (%)	Relative plating efficiency (RPE) (%)	Relative total growth (RTG) (%)
NC1	3.00	10.23	9.12	15.55	100.00	103.91	100.00	100.00
NC2	3.00	9.89	9.21	15.18				
PC	3.00	8.67	7.27	10.51	68.37	61.30	59.00	40.33
NE’X Glue1	3.00	9.76	9.05	14.72	95.81	87.96	93.99	90.05
NE’X Glue2	3.00	9.88	8.96	14.75	96.02	85.35	91.20	87.58

*PC* positive control, *NC* negative control.

**Table 8:** Toxicity data, without metabolic activation, 24 h exposure.

Sample	Number of seeded cells (× 10^5^)	Number of cells after 24 h treatment (× 10^5^)	Number of cells after 48 h treatment (× 10^5^)	Total suspension growth	Relative suspension growth (RSG) (%)	Plating efficiency (%)	Relative plating efficiency (RPE) (%)	Relative total growth (RTG) (%)
NC1	2.00	11.18	9.73	130.95	100.00	90.82	100.00	100.00
NC2	2.00	10.95	9.62	129.57				
PC	2.00	8.58	8.72	72.67	55.79	64.87	71.43	39.85
NE’X Glue1	2.00	10.55	9.61	115.58	88.73	82.85	91.23	80.95
NE’X Glue2	2.00	10.67	9.76	112.34	86.25	85.35	93.99	81.06

*PC* positive control, *NC* negative control.

**Table 9:** Mutagenicity data, without metabolic activation, 4 h exposure.

Sample	Number of large colonies	Number of small colonies	Mutant frequency (× 10^−6^)	Small colonies (%)	Small colonies mutant frequency (× 10^−6^)	Mutagenicity
NC1	78.00	2.00	128.82	2.50	3.22	N/A
NC2	64.00	2.00	97.72	3.03	2.96	N/A
PC	130.00	60.00	518.83	31.58	163.84	Mutagenic
NE’X Glue1	82.00	3.00	127.60	3.53	4.50	Not mutagenic
NE’X Glue2	70.00	4.00	119.86	5.41	6.48	Not mutagenic

*NC* negative control, *PC* positive control.

**Table 10:** Mutagenicity data, 4 h exposure, with metabolic activation.

Sample	Number of large colonies	Number of small colonies	Mutant frequency (× 10^−6^)	Small colonies (%)	Small colonies mutant frequency (× 10^−6^)	Mutagenicity
NC1	85.00	2.00	128.94	2.30	2.96	N/A
NC2	80.00	2.00	111.01	2.44	2.71	N/A
PC	54.00	88.00	376.58	61.97	233.38	Mutagenic
NE’X Glue1	74.00	6.00	132.80	7.50	9.96	Not mutagenic
NE’X Glue2	68.00	6.00	125.40	8.11	10.17	Not mutagenic

*NC* negative control, *PC* positive control.

**Table 11:** Mutagenicity data, 24 h exposure, without metabolic activation.

Sample	Large colonies number	Small colonies number	Mutant frequency (× 10^−6^)	Small colonies (%)	Small colonies mutant frequency (× 10^−6^)	Mutagenicity
NC1	78.00	2.00	122.97	2.50	3.07	N/A
NC2	64.00	2.00	108.83	3.03	3.30	N/A
PC	130.00	90.00	655.71	40.91	268.25	Mutagenic
NE’X Glue1	82.00	3.00	150.99	3.53	5.33	Not Mutagenic
NE’X Glue2	70.00	4.00	125.40	5.41	6.78	Not Mutagenic

*NC* negative control, *PC* positive control.

### Endotoxins

**Figure 3:**
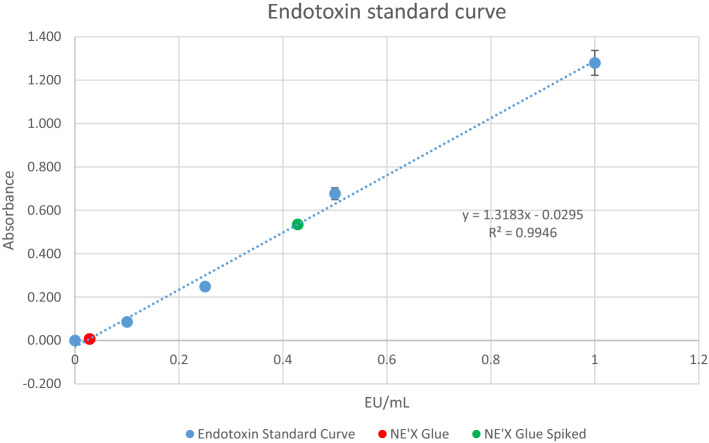
Endotoxins concentration standard curve and results.

NE'X Glue endotoxin concentration was measured as 0.028 EU/ml for a nonspiked sample and 0.428 EU/ml for a spiked sample. The calculated endotoxin content per maximal size of the device is 0.29 EU. Results and standard curve are presented in Fig. 3.

### Sensitization

Each day animals were observed. No signs of abnormalities were spotted. None of the evaluated animals lost 10% or more body weight, and none of the animals died. Furthermore, 0% of sensitization potential was observed in test sample extracts and solvent controls. Therefore, the sensitization grade for each test group (sodium chloride and cottonseed oil extracts) and solvent controls group was 0 as per Mangusson and Kligman scale.

### Intracutaneous reactivity

The Primary Irritation Index (PII) for both extracts was determined by subtracting the control group's total Primary Irritation Score from the total Primary Irritation Score of the study group. For cottonseed oil and sodium chloride extracts of NE'X Glue Surgical Adhesive, the Primary Irritation Index was calculated as 0.48 and 0.00 respectively. Results are presented in Table 12. Samples for which Total Primary Irritation Score of less than 1 are considered non irritating.

**Table 12:** Intracutaneous reactivity results.

Group	24 h after injection		48 h after injection		72 h after injection		Total Primary Irritation Score
	Average erythema	Average oedema	Average erythema	Average oedema	Average erythema	Average oedema	
NE’X Glue—Sodium Chloride	0	0	0	0	0	0	0
Solvent control—Sodium Chloride	0	0	0	0	0	0	0
NE’X Glue—Cottonseed oil	0	0.3 (3)	0.4 (6)	0.3 (3)	0.1 (3)	0.2	0.4 (8)
Solvent control—Cottonseed oil	0	0	0	0	0	0	0

### Acute systemic toxicity

**Figure 4:**
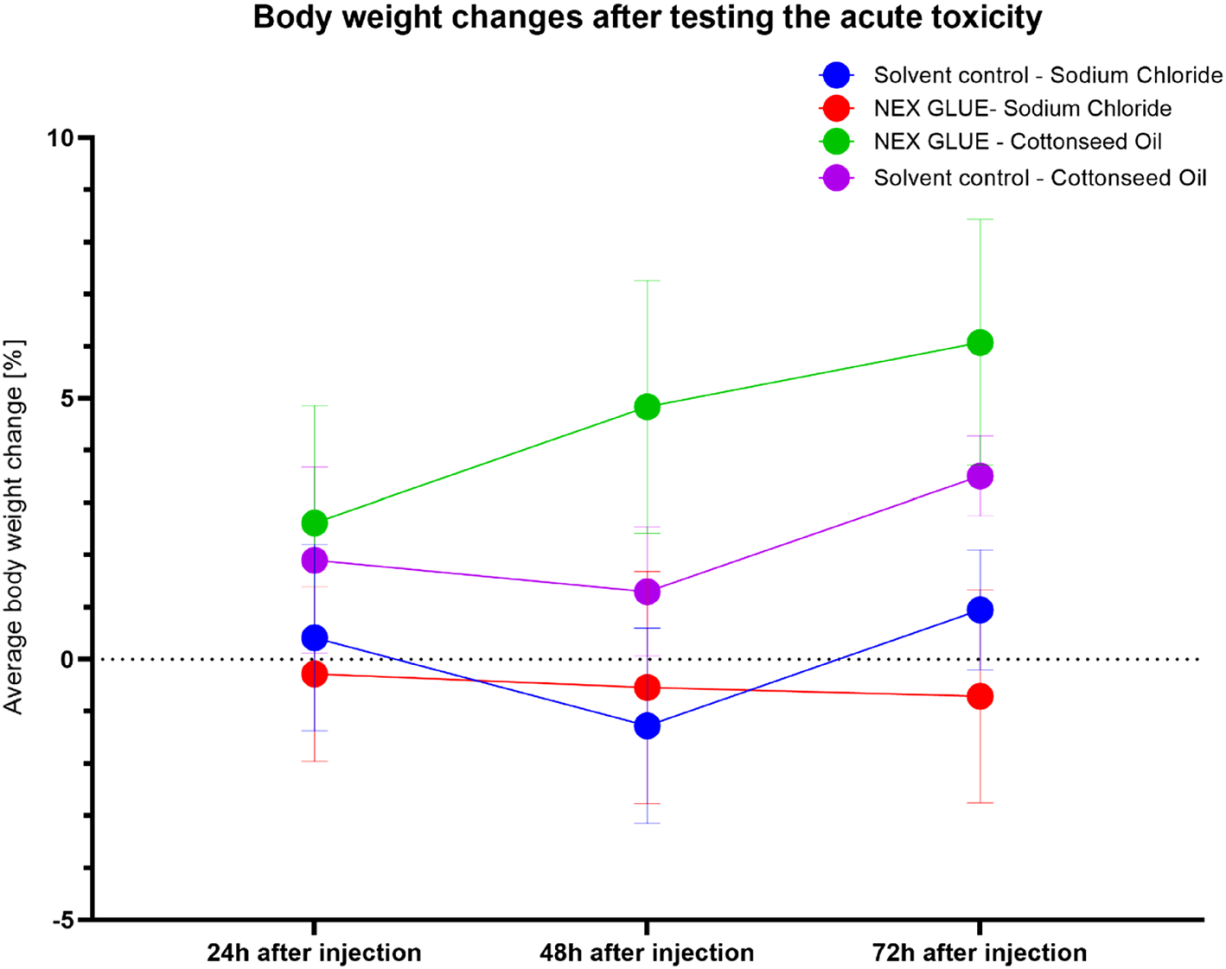
Body weight changes.

No control nor test animals showed overt signs of toxicity, listed in Table C.1, Annex C—Common clinical signs and observations, ISO 10993-11, at any observation time points^20^. None of the animals treated with the test sample showed significantly higher biological reactivity during the observation period than in the control group. None of the animals died, and none of the animals' lost 10% or more body weight. Body weight changes are presented in Fig. 4.

### Subchronic toxicity combined with implantation

**Figure 5:**
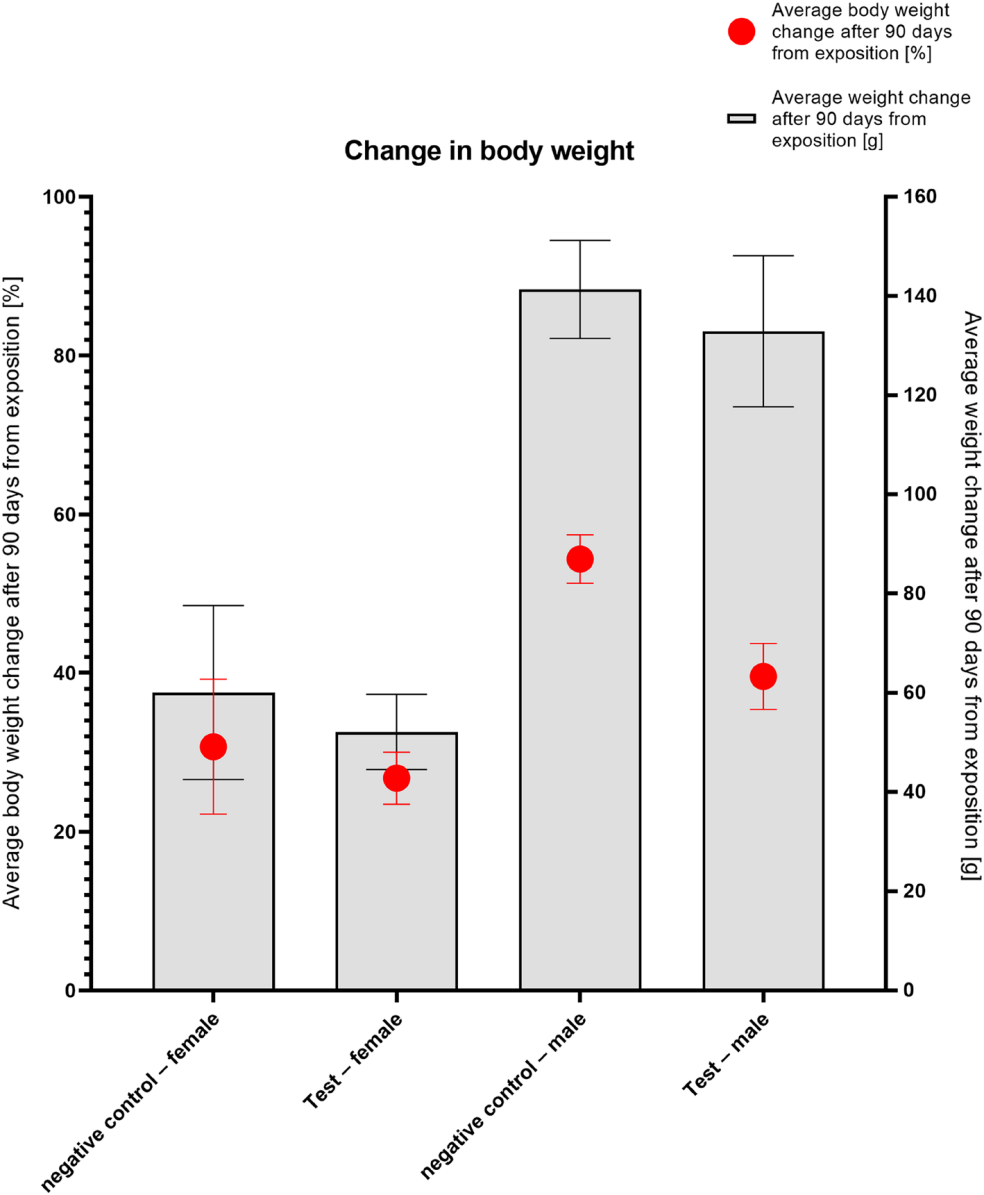
Changes in body weight.

No control nor test animals showed overt signs of toxicity, listed in Table C.1, Annex C—Common clinical signs and observations, ISO 10993-11, at any observation time points^20^. None of the animals treated with the test sample showed significantly higher biological reactivity during the observation period than in the control group. None of the animals died, and none of the animals' lost 10% or more body weight. Body weight changes are presented in Fig. 5.

#### Gross necropsy findings

**Figure 6:**
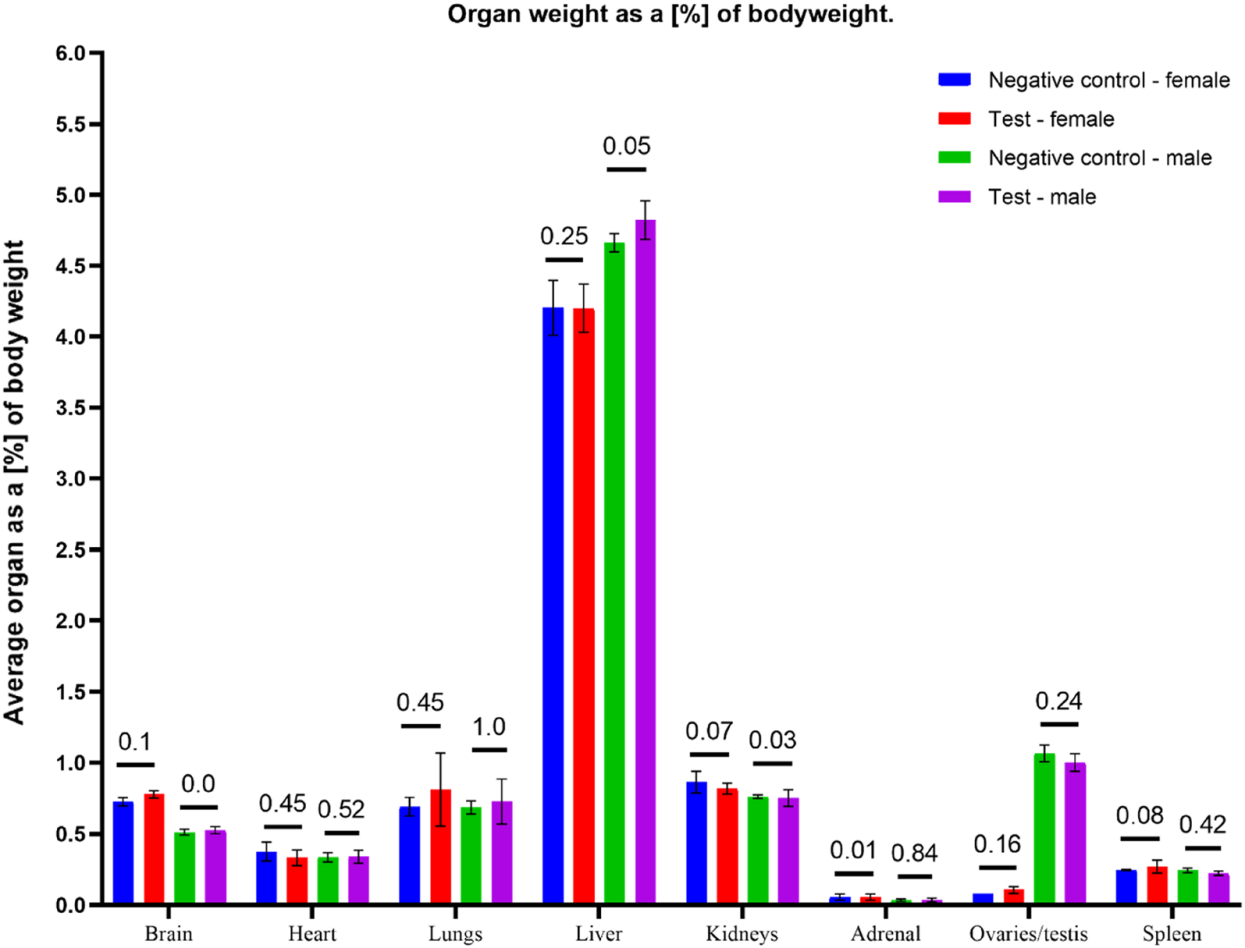
Organ weight as a (%) of body weight. Statistically significant differences were observed during the comparison of the control and test group of males' brains and kidneys, but the results did not impact the clinical picture of animals. The macroscopic and microscopic observations did not show any anomalies. No other statistically significant differences between the test and the control group were observed.

**Figure 7:**
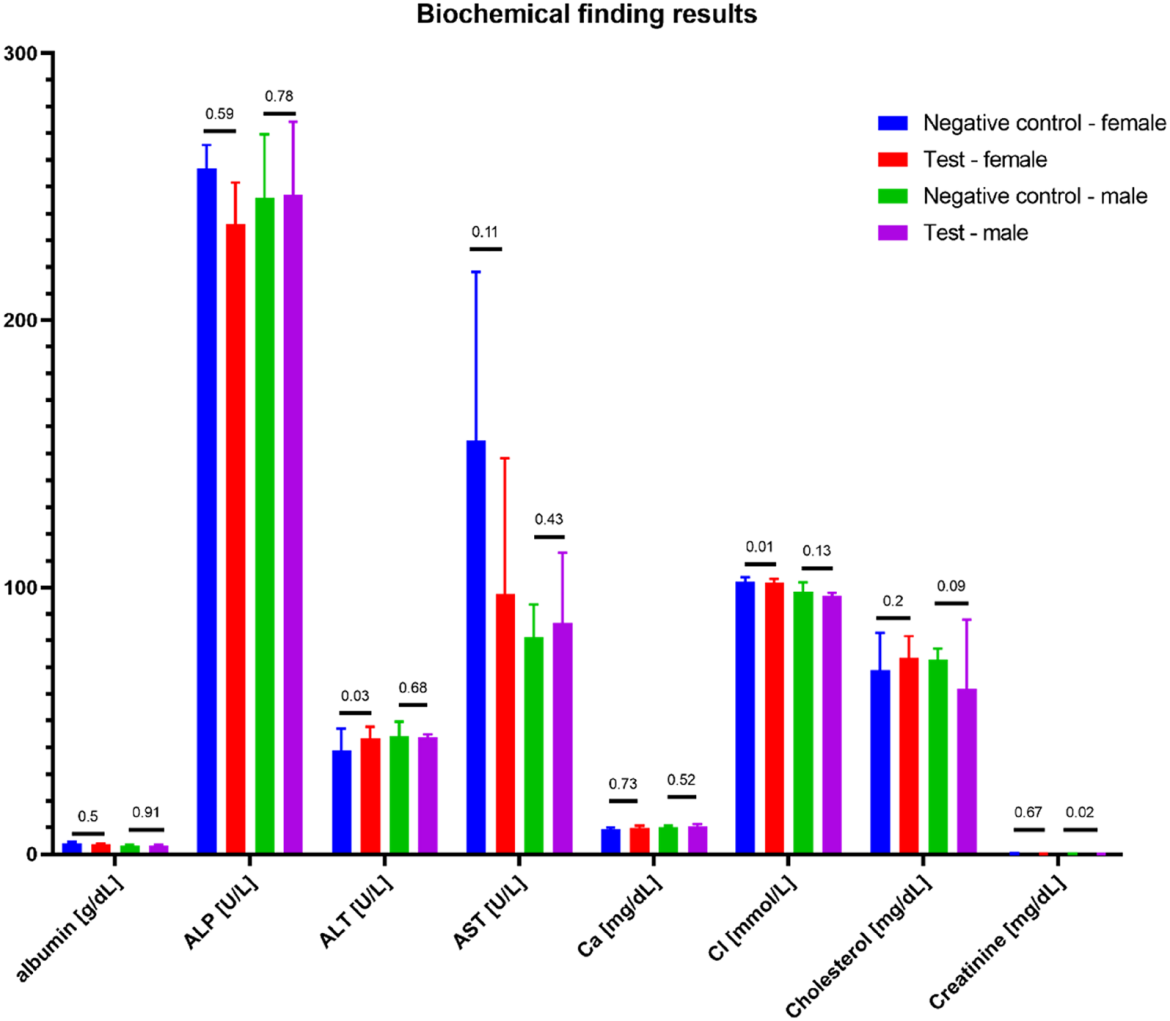
Biochemical finding results. Statistically, significant differences were observed in Cl and ALT levels between female rats' control and test group. Also, differences were observed in creatinine levels in male rats between test and control groups. However, the difference did not impact the clinical status of the animals. No other statistically significant differences were observed among the remaining parameters.

**Figure 8:**
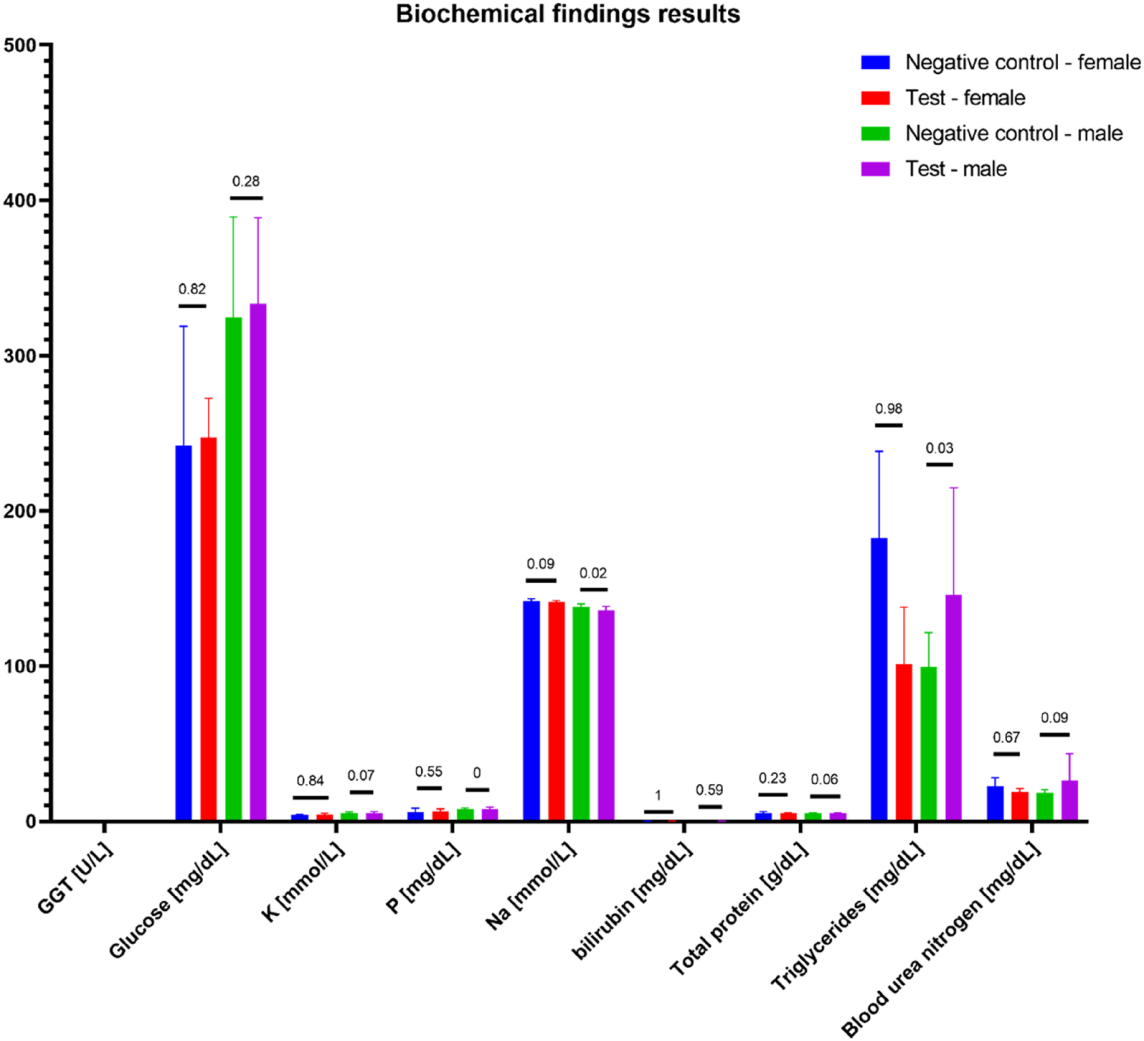
Biochemical findings results. Statistically, significant differences were observed in triglycerides, Na, and P levels between the control and test group of male rats. However, the difference did not impact the clinical status of the animals. No other statistically significant differences were observed among the remaining parameters.

**Figure 9:**
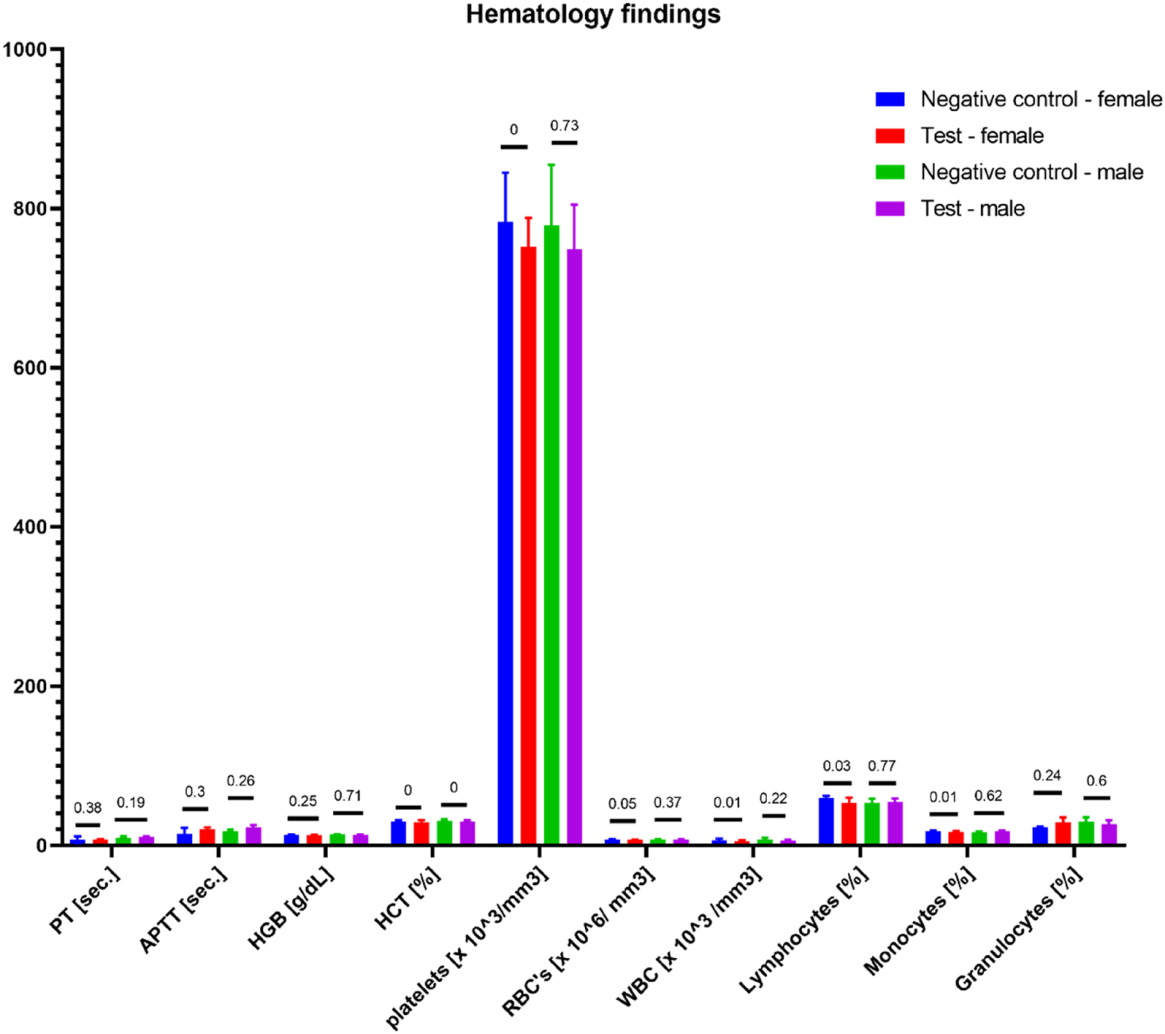
Hematology findings. Statistically significant differences were observed in HCT, WBC, lymphocytes, and monocytes between the control and test group of female rats as well as HCT in male rats. However, the difference did not impact the clinical status of the animals. No other statistically significant differences were observed among the remaining parameters.

**Figure 10:**
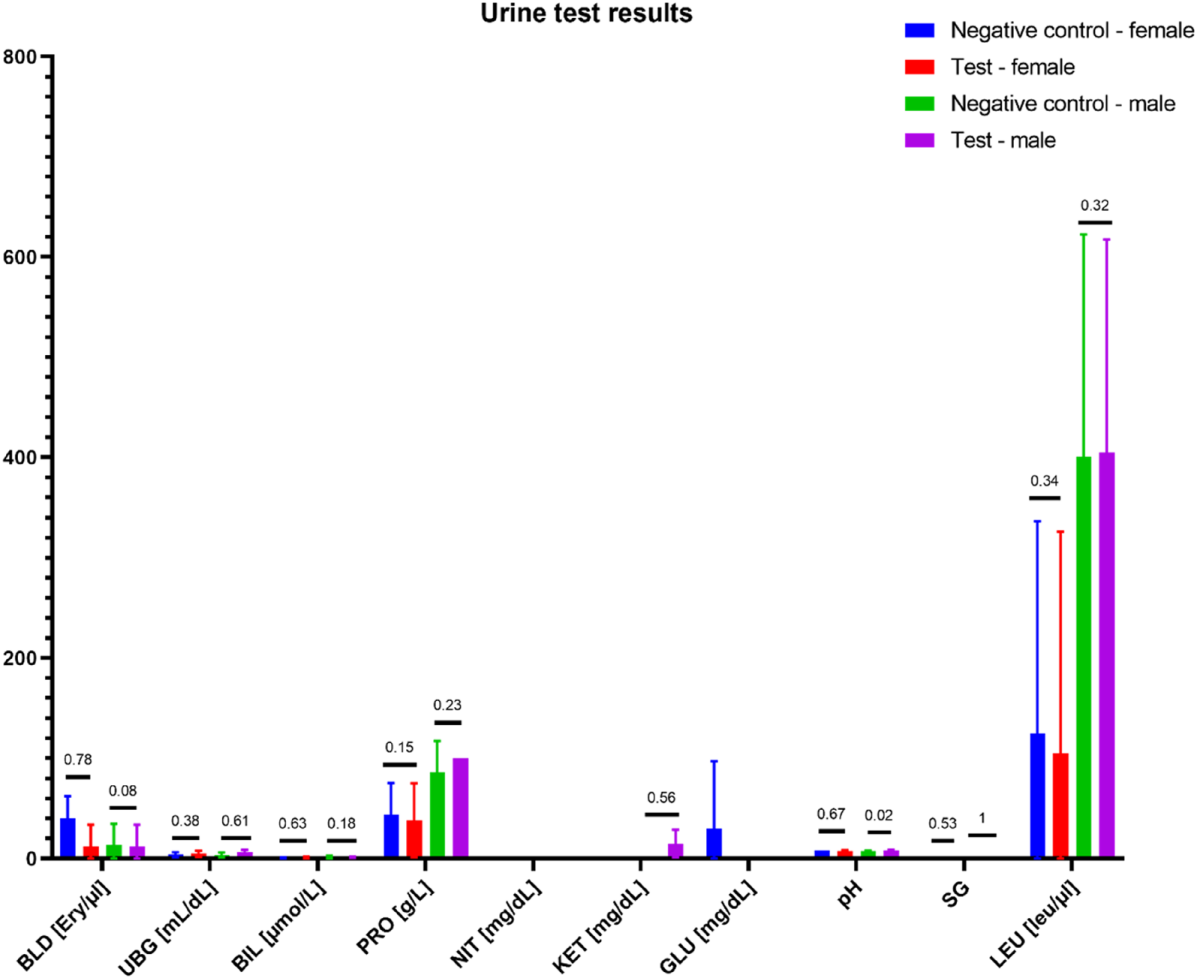
Urine test results. Statistically significant differences were observed in urine pH between the control and test group of male rats. However, the difference did not impact the clinical status of the animals. No other statistically significant differences were observed among the remaining parameters.

During gross necropsy, no abnormalities have been observed. Subcutaneous implantation sites and the surrounding tissues did not show any anomalies. During gross necropsy, kidneys, lungs, liver, heart, brain, ovaries/testis, and spleen were weighed. Organ weight was given as the % of the animal's body weight. Test results are presented in Figs. 6, 7, 8, 9 and 10.

Limited analysis was conducted instead of full histopathology as per ISO 10993-11^20^. Briefly, two representative animals were chosen from both the study and control groups. Following organs were examined: bone, bone marrow, heart, liver, lungs, kidneys, ovaries/testis, and spleen. During the histopathology evaluation, no anomalies were found. On the microscopic level, the organ's structure was normal, with no signs of apoptosis of structural cells of individual organs. No significant differences between the study and control groups were observed. Results of histological evaluation are presented in Table 13.

**Table 13:** Results of histological evaluation of the implantation place.

	Control group	Study group
	**Cell type/response**	
Polymorphonuclear cells	1	1.6
Lymphocytes	4	2.95
Plasma cells	1	0.8
Macrophages	4	3.45
Giant cells	0	0.4
Necrosis	0	0.3
	**Tissue response**	
Neovascularization	2	1.75
Fibrosis	3	1.95
Subcutaneous changes	4	2.6
Fatty infiltrate	1	0.4
Muscular layer infiltration	4	2
Total	34	27.07
Sub total	34	27.07
Average	34	27.07

According to the ISO 10993-6, double “cell-type response” scores and “tissue response scores” were summarized and divided by the number of groups to calculate the average score for study and control groups^19^. The “final reaction rating” was determined by subtracting the average negative control score from the average tested sample score. The rating of reaction for NE'X Glue was − 6.3 (0), which is classified as minimal or no reaction.

### Pyrogenicity assay

No rabbit showed individual temperature rise higher or equal to 0.6 °C above its initial temperature. Pyrogenicity test results are presented in Table 14.

**Table 14:** Results of the pyrogenicity test.

Rabbit No.	Rabbit weight (g)	Volume injected (ml)	Initial temperature (°C)	Maximal temperature (°C)	Temperature rise [°C]	Total temperature rise (°C)
1	3340	33.4	38.9	39	0.1	0.3
2	3845	38.45	38.9	39	0.1	
3	3385	33.85	38.8	38.9	0.1	

Summarized results of NE'X Glue biocompatibility testing are presented below in Table 15.

**Table 15:** NE’X Glue biocompatibility testing summary.

Test performed	Extract(s)	Test and control(s) Positive control (+) Negative control (−)	NE’X Glue results
Chemical characterization ICP-MS ISO 10993-18	Water for injection	Water for injection (−)	Elements < LOD
Chemical characterization Headspace GC–MS ISO 10993-18	Water for injection	Water for injection (−)	< AET
Cytotoxicity ISO 10993-5	MEM	DMSO (+) HDPE (−)	No cytotoxicity
Genotoxicity (MLA) ISO 10993-3	F5	F5 (−) without S9: methylmethansulfonate (+) with S9: benzo[a]pyrene (+)	No mutagenic potential
Genotoxicity (Ames) ISO 10993-3	Water for injection	Water for injection (−) without S9: 2-NF, 4-NQO, N4-ACT, 9-AA (+) with S9: 2-AA, 2-AF(+)	No mutagenic potential
Endotoxins ISO 10993-11	Water for injection	Water for injection (−)	< 0.3 EU/10 ml device
Sensitization (GPMT) ISO 10993-10	Sodium chloride Cottonseed oil	Sodium chloride (−) Cottonseed oil (−)	No sensitization
Intracutaneous Reactivity ISO 10993-10	Sodium chloride Cottonseed oil	Sodium chloride (−) Cottonseed oil (−)	No irritation
Acute Systemic Toxicity ISO 10993-11	Sodium chloride Cottonseed oil	Sodium chloride (−) Cottonseed oil (−)	No signs of toxicity
Subchronic toxicity with implantation ISO 10993-6 ISO 10993-11	Direct implantation	BioGlue surgical adhesive (−)	No signs of subchronic toxicity No difference in tissue reaction
Material mediated pyrogenicity ISO 10993-11	Sodium chloride	Sodium chloride (−)	No pyrogenicity

## Discussion

The primary objective of biocompatibility assessment is to protect people from potential biological risks arising from the use of medical devices. The ISO 10993 family standards provide guidance on the biological evaluation of medical devices in the risk management process as part of each medical device's overall evaluation and development. The evaluation process contains multiple steps, and its sole purpose is to predict if the tested device will be safe for clinical usage. Since the medical devices vary in terms of their intended use, complexity, and associated risk, the set of tests to be performed has to be chosen appropriately.

NE'X Glue is biodegraded and resorbed by the body after more than 24 months. Therefore, NE'X Glue is classified as an implant that contacts tissue for a long time (more than 30 days) as per Table A.1 in ISO 10993-1^7,21^. As a potentially high-risk device, the performed tests must be able to evaluate all the necessary endpoints, as per ISO 10993-1. Preliminary testing of extraction conditions suitable for NE'X Glue chemical analysis showed that semi-polar and non-polar solvents cause degradation of the sample. Therefore, only water was used for chemical testing. We did not observe any compounds above AET. Based on these results, no further toxicological assessment was necessary. Also, ICP-MS analysis revealed no elements above LOD, which were lower than Parenteral PDE limits present in ICH Q3D(R1) guidelines^22^. Therefore, chemical analysis of NE'X Glue extracts showed that there is no toxicological risk associated with the composition of the studied product.

Furthermore, AMES Penta 2 assay showed that NE'X Glue does not present any mutagenic potential, with and without the presence of the S9 fraction, to any of the strains employed in the test. In order to confirm that NE'X Glue is not mutagenic, especially in the eukaryotic system, the mouse lymphoma assay (MLA) was performed. The tested product did not show any mutagenic effects in any condition tested (4 h with and without metabolic activation and 24 h without metabolic activation) and therefore should be considered non-mutagenic. The combined results of chemical, mutagenicity, and genotoxicity testing indicate that carcinogenesis risk associated with the use of NE'X Glue is negligible. The lack of substances of very high concern (SVHCs), which involve substances that are carcinogenic, mutagenic or toxic to reproduction and compounds having endocrine-disrupting properties, as well as the lack of genotoxic and mutagenic potential of NE'X Glue shows that use of this device is safe even in patients with genetic abnormalities and the occurrence of late side effects is unlikely.

Potential contamination of medical devices with endotoxins may be a severe health hazard that leads to significant short and long-term complications, including abnormal CSF distribution, acute inflammation, a decline of organ function, and disrupted humoral and cellular mediation systems^23,24^. The general limit of endotoxin for medical devices intended to be used in adults is 20 EU/device, while for procedures involving contact with cerebrospinal fluid, the limit is 2.15 EU/device^25^. The endotoxin content of the maximal size of the product was evaluated in accordance with 85. Bacterial Endotoxin Test, U.S. Pharmacopeia^26^ is 0.29 EU per 10 ml device. The results show that the NE'X Glue is not only significantly below the limit for medical devices but also can be used in procedures involving contact with the cerebrospinal without hesitation. Furthermore, in vitro results were confirmed with Rabbit Pyrogen Study according to the ISO 10993-11^20^, which proved that there is no pyrogenic potential.

The cytotoxicity analysis performed according to the ISO 10993-5 showed that NE'X Glue is no cytotoxic to L-929 mouse fibroblast cells in MEM elution assay. This assay is of crucial importance due to of presence of aldehyde in the studied medical device. Exposure of tissue and cells to aldehyde can lead to irritation, sensitization and/or necrosis. Results show that the aldehyde solution effectively crosslinks the albumin and does not leak from the adhesive. This indicates that there is no risk of tissue necrosis or inflammation associated with the clinical use of evaluated surgical adhesive. The in vitro results of NE'X Glue risks associated with irritation and sensitization potential were further confirmed with in vivo studies. The potential to cause an allergic response was evaluated using the Guinea Pig Maximization Test, while the irritating potential was studied with the Intracutaneous Reactivity test. Results of both tests showed that the is no sensitizing nor irritating potential associated with the use of NE'X Glue. Acute systemic toxicity testing results provide information on immediate risks associated with using a medical device, while subchronic systemic toxicity testing combined with implantation provides data on long-term contact. The results of in vivo studies showed no immediate or prolonged risk of toxicity associated with the use of NE'X Glue Surgical Adhesive even though the dose was more than 10× of the human dose, indicating that it can be used regardless of the patient's current condition.

## Conclusion

In conclusion, NE'X Glue showed very good biocompatibility and should be considered safe for use. Therefore, NE'X Glue is a new and promising surgical adhesive with plenty of potential applications.

## Supplementary Information

Supplementary Information.

## Data Availability

The datasets generated during and/or analyzed during the current study are available from the corresponding author on reasonable request. Additional information and data for chemical characterization, MLA, media compositions, AMES assay, intracutaneous reactivity, subchronic toxicity, sensitization, and pyrogenicity combined with implantation in Tables S1–Tables S21.

## Abbreviations

AET: Analytical evaluation threshold
AMES: Bacterial Reverse Mutation Test
LLNA: Local Lymph Node Assay
LNC: Lymph node cells
MLA: Mouse Lymphoma Assay
PC: Positive control
RPE: Relative plating efficiency
RSG: Relative suspension growth
RTG: Relative total growth
SI: Sensitization Index
SVOC: Semi-volatile organic compound
TEE: Total element exposure
VOC: Volatile organic compound
