# Supplementary material for: Biocompatibility of novel albumin-aldehyde surgical adhesive
Source: Sci Rep. 2022 Jul 26;12:12749. doi: 10.1038/s41598-022-16853-5 (PMC9325888; doi:10.1038/s41598-022-16853-5)
Supplement: Supplementary file 1 — Supplementary Information. [file 41598_2022_16853_MOESM1_ESM.docx]

**Additional Materials and Methods information**

**Additional information for Chemical characterization**

GC MS study: QP2010 Ultra gas chromatograph and QP-5000 mass spectrometer (Shimadzu) were used for analysis

ICP MS study: NexION 300D (Perkin Elmer). As per ICH Q3D (R1), the concentration of the following elements was examined: Cd, Pb, As, Hg, Co, V, Ni, Tl, Au, Pd, Ir, Os, Rh, Ru, Se, Ag, Pt, Li, Sb, Ba, Mo, Cu, Sn, Cr

For the analysis of VOCs the Analytical Evaluation Threshold (AET) was calculated according to the following formula:

$$AET \left( \frac{\mu g}{ml} \right)= \frac{DBT* \frac{A}{BC}}{UF}$$

where:

A – number of devices extracted

B – volume of the extract

C – clinical exposure to medical device per day

DBT – the dose based threshold (TTC)

UF – uncertainty factor (as per ISO 10993-18 value of 2 for semiquantative analysis is assumed)

**Additional information for MLA study.**

In short, mycoplasma-free L5178Y TK+/-3.7.2C cells were cultured (37±1°C, 5±1% CO_2_, 95% humidity) in the F10 medium to the sufficient number and cleansed using THMG for 1 day and then THG for 2 days. The cleansed cells were then used in the experiment. 6 * 10^5^ (for 4h treatment) or 4 * 10^5^ (for 24h treatment) cells were exposed to the 10 ml of the 100% sample extract (worst case scenario), appropriate positive control, or negative control. The cells treatment was performed for 4h with and without the presence of 1% liver Aroclor-induced S9 fraction and for 24h without S9 fraction in 37±1°C, 5±1% CO_2_, 95% humidity. For each condition, duplicate test sample, duplicate negative control, and one positive control were prepared. After the 4h treatment, the cells were centrifuged and washed twice with fresh medium and then resuspended in the 20 ml of the F10 medium. After additional 20 hours, the cells were counted and resuspended in a fresh F10 medium at the concertation of 2 * 10^5^. The cells were incubated for 24h (37±1°C, 5±1% CO_2_, 95% humidity) and recounted. After the 24h treatment, the cells were counted, washed twice, and resuspended in F10 fresh medium at the concertation of 2 * 10^5^. The cells were incubated for 24h (37±1°C, 5±1% CO_2_, 95% humidity) and recounted. The number of cells counted was used to calculate total suspension growth (TSG) according to ISO 10993-33. After the expression period, the cell's relative plating efficiency (RPE; percentage plating efficiency of the test group in relation to the negative control) was determined by seeding a statistical number of 1,6 cells/well in two 96-well plates in F20 medium. The cells were incubated for 14 days at 37±1°C in the humidified atmosphere in the presence of 5% CO_2_. Analysis of results was based on the number of cultures without cell growth compared to the total number of cultures seeded. Relative suspension growth and relative total growth (RSG and RTG; RTG = RSG x RPE / 100) of the treated cell cultures were calculated according to the ISO 10993-33. Additionally, cultures were seeded in a selective medium. Cells from each experimental group were seeded in four 96-well plates at a density of 2000 cells/well in 200 µl selective F20 medium with TFT. The plates were scored after an incubation period of 14 days at 37±1°C in the humidified atmosphere in the presence of 5% CO_2_. Small colonies were counted separately. Small colonies are defined as less than a quarter of the diameter of the well, while large colonies are more than a quarter of the diameter of the well. The mutation frequencies were calculated from the data obtained from cultures used for the plaiting efficiency (cultures with non-selective medium) and those used for selection (cultures with selective medium) according to the following formula:

$$Mutant frequency per {10}^{6}cells= \frac{\frac{-ln(empty wells of mutant selection plates/384)}{2000}}{\frac{-\ln(empty wells of non selection plates/192)}{1,6}}*{10}^{6}$$

Table S1. MLA acceptance criteria

| **Negative control** | |
| --- | --- |
| Mutant frequency (MF) of negative control | 35 to 140 TFT resistant mutants per 10^6^ cells |
| Plating efficiency of the negative control | between 60% and 120% |
| Total suspension growth (TSG) | 8-32 for 4h treatment and 32-180 for 24h treatment |
| **Positive control** | |
| 4h treatment | ≥ 100 mutants over negative control  or  small colony MF ≥ 300 (*10-6) over negative control |
| 24h treatment | MF ≥ 300 (*10-6) over negative control with 40% small colonies  or  small colony MF ≥ 300 (*10-6) over negative control |

Table S2. Reliability check of the assay.

| Conditions | Mutant frequency of NC | Cloning efficiency of NC | Total suspension growth of NC | Positive control |
| --- | --- | --- | --- | --- |
| 4h exposure, without metabolic activation | Met criteria | Met criteria | Met criteria | Met criteria |
| 4h exposure, with metabolic activation | Met criteria | Met criteria | Met criteria | Met criteria |
| 24h exposure, without metabolic activation | Met criteria | Met criteria | Met criteria | Met criteria |

**Media compositions**

F5 medium

RPMI 1640 medium supplemented with 5% horse serum, 1% penicillin/streptomycin, 1% Pluronic F-68, and 1% sodium pyruvate.

F10 medium

RPMI 1640 medium supplemented with 10% horse serum, 1% penicillin/streptomycin, 1% Pluronic F-68, and 1% sodium pyruvate.

F20 medium

RPMI 1640 medium supplemented with 20% horse serum, 1% penicillin/streptomycin, 1% Pluronic F-68, and 1% sodium pyruvate.

Selective F20 medium with TFT

RPMI 1640 medium supplemented with 20% horse serum, 1% penicillin/streptomycin, 1% Pluronic F-68, 1% sodium pyruvate, and 3 μg/ml TFT.

THMG 100x stock

300 μg/ml Thymidine

500 μg/ml Hypoxanthine

750 μg/ml Glycine

10 μg/ml Methotrexate

THG 100x stock

300 μg/ml Thymidine

500 μg/ml Hypoxanthine

750 μg/ml Glycine

S9 mix (per sample)

S9 fraction 0.1 ml

150 mM KCl 0.1 ml

638 mM G6P 0.1 ml

33 mM NADP 0.1 ml

dH2O 0.1 ml

pH adjusted to 7 with 1 N NaOH.

For each 4h treatment with presence of S9 fraction 0.5 ml of S9 mix per 9.5 ml of cell culture medium was used resulting in the final concentration of S9 fraction at 1%.

Negative control: F5 medium with or without S9 fraction.

Positive controls: 10 μg/ml methylmethansulfonate for cultures without S9 fraction

3 μg/ml benzo[a]pyrene for cultures with S9 fraction

**Additional information for AMES assay.**

Bacteria were exposed to the 25 µl of full strength extracts of the test material as well as positive and negative controls for 135 minutes in a medium containing sufficient histidine (S. typhimurium) or tryptophan (E. coli) to support approximately two cell divisions. The volume of extract added was based on the ISO 10993-33 and kit manufacturers' documentation. After exposure, the cultures were diluted in a pH indicator medium lacking histidine or tryptophan and aliquoted into 48 wells of a 384-well plate. After two days, cells that have undergone reversion to amino acid prototrophy grow into colonies. Bacterial metabolism reduces the pH of the medium, changing the color of that well. The number of wells containing revertant colonies were counted for each group (test article and positive control) and compared to a solvent (negative) control. Samples were prepared in triplicate to allow for statistical analysis of the data. The mutagenic potential of samples was assessed directly and in the presence of 4,5% of liver Aroclor-induced S9 fraction. Baseline, fold increase over baseline value, and binomial B-value were calculated using an excel spreadsheet provided by the manufacturer. The baseline is calculated as a mean plus standard deviation of the negative control. Fold increase over baseline is calculated by dividing the mean number of positive wells for a sample by the baseline value. The binomial B-value indicates the probability that spontaneous mutation events occur. For example, a binomial B-value ≥ 0,99 indicates that chances are ≤ 1% that this Result is due to spontaneous mutation. If both fold increase ≥ 2 and binomial B-value ≥ 0,99 occur for a test sample in specific conditions (strain, +/- S9 fraction), it should be considered mutagenic. Results are presented in the Table S3.

Table S3. Positive controls list for AMES test.

| **Strain** | **Substance** | |
| --- | --- | --- |
|  | **Without S9 fraction** | **With S9 fraction** |
| *Salmonella typhimurium* TA98 | 2-nitrofluorene (2-NF) | 2-aminoanthracene (2-AA) |
| *Salmonella typhimurium* TA1535 | N4-aminocytidine  (N4-ACT) | 2-AA |
| *Salmonella typhimurium* TA1537 | 9-aminoacridine  (9-AA) | 2-AA |
| *E.coli*  WP2 uvrA[pKM101] | 4-NQO | 2-aminofluorene  (2-AF) |

**Additional information for Intracutaneous reactivity.**

Before the treatment, the fur on the each rabbit's back on both sides of the spinal column was closely clipped over a sufficiently large test area, avoiding mechanical irritation and trauma. Then, 0,2 ml of the polar (Sodium Chloride) and non-polar (Cottonseed Oil) extracts were injected intracutaneously at five sites on one side of each rabbit (New Zealand). Similarly, 0.2 ml of the polar and non-polar solvent controls were injected intracutaneously on five sites of the contralateral side of each rabbit. The animals were observed immediately after injection, 24±2, 48±2, and 72±2 hours after the treatment to evaluate the signs of local reaction. Injection sites were examined for evidence of any tissue reaction such as erythema, oedema, and eschar. Tested and control sites were scored according to the Table S4.

Table S4. The grading system for intracutaneous (intradermal) reactions.

| **Reaction** | **Numerical grading** |
| --- | --- |
| **Erythema and eschar formation** | |
| No erythema | 0 |
| Very slight erythema (barely perceptible) | 1 |
| Well defined erythema | 2 |
| Moderate erythema | 3 |
| Severe erythema (beet redness) to eschar formation preventing grading of erythema | 4 |
| **Oedema formation** | |
| No oedema | 0 |
| Very slight oedema (barely perceptible) | 1 |
| Well defined oedema (edges of area well defined by define raising) | 2 |
| Moderate oedema (edges raised approximately 1 mm) | 3 |
| Severe oedema (raised more than 1 mm and extended beyond exposure area) | 4 |

After the 72±2 h grading, all erythema grades plus oedema grades (at 24±2 h, 48±2 h, and 72±2 h) are separately summed for each test sample or blank for each animal. To calculate the score of a test sample or blank on each animal, divide each totals by 15 (3 scoring time points x 5 test or blank sample injection sites). To determine the overall mean score for each test sample and each corresponding blank, add the scores for the three animals and divide them by three. The final test sample score is obtained by subtracting the blank score from the test sample score. The acceptance criteria are met if the final test score is 1.0 or less.

**Additional information for Subchronic toxicity combined with implantation.**

Gross necropsy

After animal euthanasia, a gross necropsy was performed on all animals. The following organs were weighed (paired organs together) after dissection: adrenals, brain, lungs, heart, kidneys, liver, ovaries, spleen, testes. The organ-to-body weight ratios (relative organ weights) were calculated from the rats' absolute organ weights and the terminal body weight.

Samples of the weighed organs and the colon, lymph nodes, skin, lungs, mammary gland, peripheral nerve (sciatic), esophagus, parathyroid, pituitary, prostate, rectum, small intestines (duodenum, ileum, jejunum), sternum with bone marrow, stomach, thyroid, trachea with bronchi, urinary bladder, uterus, vagina, places of implantation and all gross lesions were preserved in a neutral aqueous phosphate-buffered 4% solution of formaldehyde. Histopathologic analysis from organs: brain, lungs, heart, liver, kidneys, adrenals, ovaries/testis, sternum, muscle, the skin was conducted on 5 μm sections of paraffin-embedded tissues, stained with hematoxylin and eosin, of the preserved organs from two representative animals per sex from control and test group by light microscopy. Each place of subcutaneous implantation was examined under a microscope and evaluated based on the guidelines provided in Table S5.

Table S5. Guidelines of histological evaluation system of place of implantation – tissue response

| **Histologic feature** | **Score** | | | | |
| --- | --- | --- | --- | --- | --- |
|  | **0** | **1** | **2** | **3** | **4** |
| Inflammatory cell type/response — Polymorphonuclear cells — Lymphocytes | 0 | Rare, 1 to 5/hpf ^a^ | Rare,5 to 10/hpf ^a^ | Moderate infiltrate | Marked infiltrate |
| Plasma cells |  |  |  |  |  |
| Macrophages/gitter cells |  |  |  |  |  |
| Multinucleated giant cells MGC) | 0 | Rare, 1 to 2/hpf | Rare, 3 to 5/hpf |  |  |
| Necrosis | 0 | Minimal | Mild | Moderate infiltrate | Marked |
| Neovascularization | 0 | Minimal capillary proliferation, focal, 1 to 3 buds | Groups of 4 to 7 capillaries with supporting fibroblastic structures | Broad band of capillaries with supporting fibroblastic structures | Extensive band of capillaries with supporting fibroblastic structures |
| Fibrosis | 0 | Narrow band | Moderately thick band | Thick band | Extensive band |
| Astrocytosis/fatty infiltration |  |  |  |  |  |
| ^a^ hpf=high-powered (400x) field. | | | | | |

Table S6. Reaction to implantation rating.

| Grade | Classification |
| --- | --- |
| 0.0 – 2.9 | Minimal or no reaction |
| 3.0 – 8.9 | Slight reaction |
| 9.0 – 15.0 | Moderate reaction |
| 15.1 | Severe reaction |

Table S7. Organ weight as a [%] of bodyweight.

| **Group** | **Average body weight [g]** | **Average organ as a [%] of body weight** | | | | | | | |
| --- | --- | --- | --- | --- | --- | --- | --- | --- | --- |
|  |  | Brain | Heart | Lungs | Liver | Kidneys | Adrenal | Ovaries / testis | Spleen |
| negative control – female | 262.13 | 0.76 | 0.33 | 0.86 | 3.70 | 0.75 | 0.06 | 0.12 | 0.27 |
| negative control – male | 405.92 | 0.59 | 0.29 | 0.67 | 3.86 | 0.69 | 0.03 | 0.97 | 0.25 |
| Test – female | 249.08 | 0.76 | 0.35 | 0.81 | 3.85 | 0.82 | 0.04 | 0.10 | 0.30 |
| Test – male | 478.00 | 0.48 | 0.30 | 0.67 | 4.26 | 0.76 | 0.03 | 0.91 | 0.24 |

Table S8. Organ weight: a statistical comparison of the control group with the test group – P-value results.

| **Sex** | **Brain** | **Heart** | **Lungs** | **Liver** | **Kidneys** | **Adrenal** | **Ovaries/testis** | **Spleen** |
| --- | --- | --- | --- | --- | --- | --- | --- | --- |
| female | 0 ,10 | 0 ,45 | 0 ,45 | 0 ,25 | 0 ,07 | 0 ,01 | 0 ,16 | 0 ,08 |
| male | 0 ,00 | 0 ,52 | 1 ,00 | 0 ,05 | 0 ,03 | 0 ,84 | 0 ,24 | 0 ,42 |

Table S9. Biochemical finding results.

| **Group** | **albumin** | **ALP** | **ALT** | **AST** | **Ca** | **Cl** | **Cholesterol** | **Creatinine** |
| --- | --- | --- | --- | --- | --- | --- | --- | --- |
|  | [g/dL] | [U/L] | [U/L] | [U/L] | [mg/dL] | [mmol/L] | [mg/dL] | [mg/dL] |
| negative control – female | 3.87 | 177.80 | 56.10 | 110.10 | 10.15 | 99.40 | 74.00 | 0.25 |
| negative control – male | 3.44 | 159.10 | 56.30 | 108.30 | 9.94 | 100.60 | 67.40 | 0.05 |
| Test – female | 3.84 | 189.70 | 70.70 | 138.20 | 10.02 | 102.40 | 81.80 | 0.30 |
| Test – male | 3.47 | 165.40 | 58.50 | 126.10 | 10.07 | 99.20 | 85.70 | 0.18 |

Table S10. Biochemical findings: a *s*tatistical comparison of the study group with the control group – P-value.

| **Sex** | **albumin** | **ALP** | **ALT** | **AST** | **Ca** | **Cl** | **Cholesterol** | **Creatinine** |
| --- | --- | --- | --- | --- | --- | --- | --- | --- |
| female | 0.5 | 0.59 | 0.03 | 0.11 | 0.73 | 0.01 | 0.2 | 0.67 |
| male | 0.91 | 0.78 | 0.68 | 0.43 | 0.52 | 0.13 | 0.09 | 0.02 |

Table S11. Biochemical findings results.

| **Group** | **GGT*** | **Glucose** | **K** | **P** | **Na** | **bilirubin** | **Total protein** | **Triglycerides** | **Blood urea nitrogen** |
| --- | --- | --- | --- | --- | --- | --- | --- | --- | --- |
|  | [U/L] | [mg/dL] | [mmol/L] | [mg/dL] | [mmol/L] | [mg/dL] | [g/dL] | [mg/dL] | [mg/dL] |
| negative control – female | * | 253.70 | 4.81 | 5.56 | 138.30 | 0.30 | 5.62 | 100.30 | 22.66 |
| negative control – male | * | 298.80 | 4.94 | 6.68 | 139.30 | 0.22 | 5.67 | 95.90 | 19.12 |
| Test – female | * | 260.30 | 4.75 | 5.25 | 140.30 | 0.31 | 5.80 | 102.10 | 24.24 |
| Test – male | * | 272.00 | 4.42 | 5.19 | 137.40 | 0.24 | 5.93 | 141.90 | 20.47 |

*level undetected or very low

Table S12. Biochemical test: a statistical comparison of the study group with the control group – P-value.

| **Sex** | **GGT** | **Glucose** | **K** | **P** | **Na** | **Bilirubin** | **Total protein** | **Triglycerides** | **Blood urea nitrogen** |
| --- | --- | --- | --- | --- | --- | --- | --- | --- | --- |
| female | NA | 0.82 | 0.84 | 0.55 | 0.09 | 1 | 0.23 | 0.98 | 0.67 |
| male | NA | 0.28 | 0.07 | 0 | 0.02 | 0.59 | 0.06 | 0.03 | 0.09 |

Table S13. Hematology findings.

| **Group** | **PT** | **APTT** | **HGB** | **HCT** | **platelets** | **RBC's** | **WBC** | **Lymphocytes** | **Monocytes** | **Granulocytes** |
| --- | --- | --- | --- | --- | --- | --- | --- | --- | --- | --- |
|  | [sec.] | [sec.] | [g/dL] | [%] | [x 10^3/mm3] | [x 10^6/ mm3] | [x 10^3 /mm3] | [%] | [%] | [%] |
| negative control – female | 9.46 | 21.35 | 13.36 | 24.96 | 631.00 | 7.22 | 2.84 | 58.36 | 15.99 | 25.66 |
| negative control – male | 11.18 | 28.46 | 13.79 | 25.29 | 851.80 | 7.79 | 6.72 | 58.72 | 15.59 | 25.62 |
| Test – female | 9.24 | 19.99 | 13.82 | 35.11 | 804.70 | 7.57 | 4.66 | 54.25 | 17.94 | 27.81 |
| Test – male | 10.45 | 21.48 | 13.65 | 32.00 | 859.40 | 7.95 | 8.09 | 57.98 | 15.25 | 26.77 |

Table S14. Hematology test: a statistical comparison of the study group with the control group – P-value.

| **Sex** | **PT** | **APTT** | **HGB** | **HCT** | **platelets** | **RBC's** | **WBC** | **Lymphocytes** | **Monocytes** | **Granulocytes** |
| --- | --- | --- | --- | --- | --- | --- | --- | --- | --- | --- |
| female | 0.38 | 0.3 | 0.25 | 0 | 0 | 0.05 | 0.01 | 0.03 | 0.01 | 0.24 |
| male | 0.19 | 0.26 | 0.71 | 0 | 0.73 | 0.37 | 0.22 | 0.77 | 0.62 | 0.6 |

Table S15. Urine test results.

| **Group** | **BLD** | **UBG** | **BIL** | **PRO** | **NIT** | **KET** | **GLU** | **pH** | **SG** | **LEU** |
| --- | --- | --- | --- | --- | --- | --- | --- | --- | --- | --- |
|  | [Ery/µl] | [ml/dl] | [µmol/l] | [g/l] | [mg/dl] | [mg/dl] | [mg/dl] |  |  | [leu/µl] |
| negative control – female | 7.00 | 2.60 | 0.30 | 48.00 | 0.00 | 0.00 | 0.00 | 8.10 | 1.01 | 25.00 |
| negative control – male | 21.00 | 3.00 | 0.50 | 58.00 | 0.00 | 5.00 | 0.00 | 7.70 | 1.02 | 362.50 |
| Test – female | 5.00 | 3.43 | 0.20 | 25.00 | 0.00 | 0.00 | 0.00 | 8.00 | 1.01 | 22.50 |
| Test – male | 6.00 | 3.56 | 0.20 | 138.00 | 0.00 | 2.50 | 0.00 | 8.40 | 1.02 | 450.00 |

Table S16. Urine test: a statistical comparison of the study group with the control group – P-value.

| **Sex** | **BLD** | **UBG** | **BIL** | **PRO** | **NIT** | **KET** | **GLU** | **pH** | **SG** | **LEU** |
| --- | --- | --- | --- | --- | --- | --- | --- | --- | --- | --- |
| female | 0.78 | 0.38 | 0.63 | 0.15 | - | - | - | 0.67 | 0.53 | 0.34 |
| male | 0.08 | 0.61 | 0.18 | 0.23 | - | 0.56 | - | 0.02 | 1 | 0.32 |

Table S17. Change in body weight.

| **Group** | **Average body weight change after 90 days from exposition [%]** | **Average weight change after 90 days from exposition [g]** |
| --- | --- | --- |
| negative control – female | 30.70% | 60.03 |
| negative control – male | 54.35% | 141.32 |
| Test – female | 26.76% | 52.11 |
| Test – male | 39.56% | 132.88 |

**Additional information for GPMT sensitization study.**

Table S18. Magnusson and Kligman scale.

| Patch test reaction | Grading scale |
| --- | --- |
| No visible change | 0 |
| Discrete or patchy erythema | 1 |
| Moderate and confluent erythema | 2 |
| Intense erythema and/or swelling | 3 |

Table S19. Rating of sensitization potential, Mangusson and Kligman 1969.

| **Sensitization rate (%)** | **Grade** | **Classification** |
| --- | --- | --- |
| 0-8 | I | Weak |
| 9-28 | II | Mild |
| 29-64 | III | Moderate |
| 56-80 | IV | Strong |
| 81-100 | V | Extreme |

**Additional information for pyrogenicity study.**

Table S20. Criteria of acceptance for the pyrogenicity test.

| Number of rabbits | Product passes if the summary response does not exceed [°C] | The product fails if the summary response exceeds [°C] |
| --- | --- | --- |
| 3 | 1.15 | 2.65 |
| 6 | 2.80 | 4.30 |
| 9 | 4.45 | 5.95 |
| 12 | 6.60 | 6.6 |

**Additional information for Acute Systemic Toxicity study.**

Table S21. Body weight changes.

| **Group** | **Average body weight change 72h after injection [%]** |
| --- | --- |
| Solvent control -Sodium Chloride | 0.88 |
| NEX GLUE-Sodium Chloride | -1.25 |
| NEX GLUE- Cottonseed Oil | 2.48 |
| Solvent control - Cottonseed Oil | 0.77 |
